# Creating a learning environment in your practice or facility

**DOI:** 10.4102/safp.v62i1.5166

**Published:** 2020-08-12

**Authors:** Bob Mash, Jill Edwards

**Affiliations:** 1 Division of Family Medicine and Primary Care, Stellenbosch University, Cape Town, South Africa

**Keywords:** Medical education, family medicine, primary care, clinical training, learning environment, leadership, clinical trainer, clinical teaching, workplace based learning

## Abstract

Family physicians are expected to build the capacity of the primary care team and to provide clinical training to students, but often lack the educational expertise and a supportive learning environment. This article aims to outline the competencies needed to fulfil these expectations and to assist with professional development in this area. The organisational environment has a profound effect on the success of learning and issues such as adequate infrastructure, optimal staff numbers and mix, appropriate patient mix, quality of care, supportive management and organisational culture are all important. Within this organisational culture learning is often dependent on an effective clinical trainer. Clinical trainers impact learning through role modelling, facilitation of learning and providing up to date information. They need educational competencies to embed brief and effective training into clinical practice, to provide effective feedback and to engage with small group teaching and assessment. The family physician can lead the entire team and practice through developing themselves as a clinical trainer, creating supportive relationships and transforming policies and processes to support innovation and learning.

## Introduction

Family physicians are expected to build the capacity of the primary care team and to provide clinical training to students,^1^ but often lack the educational expertise and a supportive learning environment. Older postgraduate training programmes in family medicine did not systematically prepare family physicians for these roles, and even today there is a need to improve and standardise training between programmes. The gap in clinical training expertise and unsupportive learning environments contribute to the low pass rate in our national fellowship examination (currently around 45%), may impair the experiences of undergraduate students and limit the development of the primary care team. Even in private practices that may not have multidisciplinary teams or students, there is a need to create a learning environment that encourages practitioners to improve the quality of care and stay up to date through continuing professional development.

Primary care teams may include nurse practitioners, interns, community service doctors, medical officers, clinical associates and community health workers, all of whom may need capacitating. Team members may have limited preservice training or clinical experience and lack confidence. In our context there is often additional task shifting or sharing, which also requires additional capacity building. Students may include medical students, nurses, clinical associates, interns and registrars.

Creating a learning environment extends beyond the individual family physician’s educational capability to the broader workplace environment. The workplace environment includes the infrastructure, workforce, quality of care, organisational culture, processes and procedures, all of which may enable or hinder people from learning.

This article focuses on how to create an effective learning environment in your primary care practice. At the heart of this endeavour is to be an effective clinical trainer and to also lead your team and organisation to develop such an environment.

## What is an effective organisational environment?

To be considered an effective learning environment, the workplace needs to be safe for patients and staff. The culture must be caring, compassionate and provide an acceptable quality of care and experience for patients, carers and families.^2^ Providing an acceptable quality of care is dependent on many factors, such as adequate infrastructure, supplies, equipment, staff numbers and mix, clinical governance and management.^3^ An under-resourced clinical environment with insufficient resources or personnel could impede learners’ completion of clinical tasks or alternatively require the learner to perform at higher levels of responsibility than they are ready for. The environment should also provide an adequate exposure to the appropriate range of patients, conditions, communities and health professionals to achieve the learning outcomes.^4^

Learning should be a valued part of the organisational culture, with a demonstrable emphasis on quality improvement and innovation. The clinical learning environment is multiprofessional, so an effective learning culture will value and support learners from all professional groups. Organisational culture, however, is not always supportive of learning, innovation and open communication.^5^ Because the biggest influence on organisational culture comes from the leadership of the organisation, it is vital that managers support learning and training.

Students enrolled in formal training programmes are increasingly placed in the distributed district health services platform, which has not traditionally hosted students. Although this is an appropriate and positive transition, the facilities are often not used to integrating the needs of the health and educational systems. Students may be seen as a burden that take energy away from service delivery rather than as potential collaborators who can contribute to clinical care and quality improvement. Although there may be a designated trainer or coordinator for students, it is important that students learn from all types of health professionals and that there be a whole-team approach to learning.

Capacity building of staff traditionally involved sending staff away on workshops and training courses. Sometimes this is necessary, but often it undermines service delivery when large numbers of staff members are away from the facility for days at a time. Often the facility sends inappropriate people for training because they are available, rather than because they need the training. Modern approaches to training rely more and more on web-based and distance forms of training that can be completed at convenient times. In addition, there is a move towards workplace-based training offered as multiple short sessions that are more tailored to fit in with service delivery pressures. It is also important to protect dedicated time for workplace-based learning and study for students who are also essential to service delivery, such as registrars and interns.

The workplace that offers opportunities for students to participate in authentic work activities powerfully affects learning and performance.^6^ While contributing to high-quality patient care, learners should be encouraged to participate by undertaking increasingly complex responsibilities for that care and be assessed within the work context. Learning environments and cultures that optimise workloads and promote greater professional development have several key features. They facilitate task assignment appropriate to learning, foster robust communication and professionalism, and include team-building activities.

Feedback is a crucial component of the process of learning from participating in clinical experiences in the workplace, and it guides professional development. Feedback enables learners to monitor their progress, provides direction for improvement and informs learners’ self-assessments.^7^ Registrars say that the workplace culture strongly influences their willingness to seek feedback.^8^ They expect regular feedback as part of the educational process but are unlikely to seek it in a setting that seems to value clinical work over learning. The faculty perceived that the organisational culture did not support teaching, and time pressures for patient care interfered with providing feedback.

Facilities that support a learning environment would also plan space for small group learning or training and provide essential educational equipment. Facilities with large numbers of students may create a learning centre in collaboration with the educational institution that provides dedicated space for study, Internet access and small group education.

Box 1 summarises some of the key characteristics of a supportive organisational environment for learning.^9^ One of the tasks of family physicians in their roles as capacity builders and clinical trainers is to lead the organisation to become a more effective learning environment.

BOX 1 Characteristics of an effective workplace-based learning environment.

1. Adequate infrastructure, supplies, equipment, space, security to support effective service delivery and quality of care.
2. Appropriate patient mix, community orientation and engagement.
3. Optimal numbers, student-to-trainer ratio, number and range of practitioners.
4. Partnership between stakeholders with open communication.
5. Supportive organisational culture with managers who support learning and innovation.
6. Whole-team approach to learning and training.
7. Use of space and infrastructure to support learning.
8. Balancing the needs of service delivery with learning.
9. Constructive feedback on performance is welcomed.
10. Protected time and prioritisation of learning for postgraduate students.

*Source:* Mehay R. The essential handbook for GP training and education. London: Radcliffe Publishing; 2012.

Tools are available to evaluate the learning environment,^10^ and local research has also identified 12 tips for distributed health professions training.^11^ This research emphasises the importance of the relationships between stakeholders who contribute to the training and the learning environment. From the perspective of the family physician, these stakeholders might include the facility and district managers, the other health professionals, the university or educational institution and local non-profit organisations. Relationships need to build understanding, partnership, trust and access to information.

## The effective clinical trainer

**FIGURE 1:**
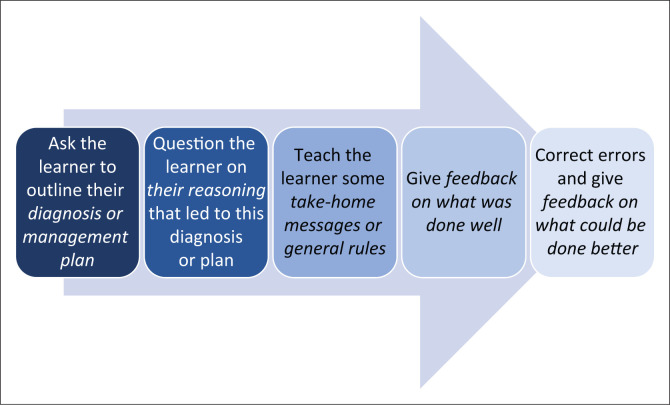
Five steps in the one-minute teacher.

Clinicians often feel that there is a lack of time to engage with teaching and training in environments with demanding clinical workloads. Although this is undoubtedly a limiting factor, there are strategies to embed brief but effective training into the clinical workplace. We need to adopt the principle of ‘any time, any place, anywhere’. Any clinical interaction then becomes an opportunity to learn something. Patients are a rich learning resource, and the teaching does not need to take place in a classroom. It can take remarkably little time, particularly if techniques such as the ‘one-minute teacher’^12^ are used (Figure 1). What then are the skills of an effective clinical trainer?

### Acting as a role model

The clinical setting provides a powerful context, and the challenge for the family physician is to make information directly relevant to the context and to illuminate the process of clinical decision-making. One way to do this is to model in your own professional behaviour and clinical practice what needs to be learnt.^13^ Role models transmit the ‘soft skills’ – values, attitudes, patterns of thought and behaviour. The family physician contributes to the environment of learning by creating mutual respect and open communication and facilitates growth by demonstrating the soft skills of dedication to their patients, a love of teaching and a caring personality. Role modelling may have a greater impact on the student than any other teaching method. Too often, inappropriate attitudes and practice are role modelled, and learners may pay more attention to what they see in practice than what is taught in the classroom.

Family physicians may act as role models not only for clinical practice, but also for educational practice. Many health professionals have been trained in top-down, authoritarian or didactic approaches to teaching, which are often less effective. How the family physician behaves as a clinical trainer will also influence the approach of others to training in the workplace.

### Facilitating learning

There is a move away from being ‘teacher–centred’, where the teacher is the transmitter of information and the focus is on what the teacher does, to being ‘student–centred’, where the focus is on the student. The more student–centred the learning, the more the role of the teacher becomes one of facilitation.^13^ The role is not to inform, but to encourage and facilitate the student to learn for themselves.

Asking questions to evoke ideas, solutions and problem-solving from the learner may be more effective than telling people the answers. Assisting learners to identify what they need to learn and to plan how they will address these needs may be more effective than prescribing what people should learn. Facilitating skilful, structured and constructive feedback, sometimes within a group, is more effective than harsh or humiliating criticism.

**FIGURE 2:**
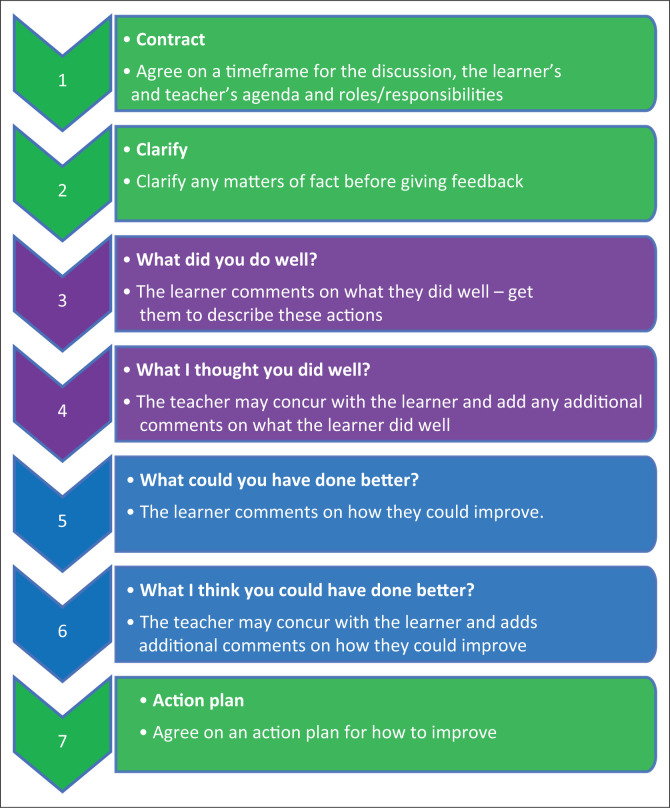
Modified Pendleton’s principles for giving feedback.

The family physician should be familiar with tools that can facilitate learning such as the mini-clinical examination tool (mini-CEX) for observing consultations,^14^ the tool for directly observed procedural skills (DOPS)^15^ or the tool for assessing brief behaviour change counselling (the ABC tool).^16^ Giving effective feedback is again an essential skill, and various models have been recommended, such as the Pendleton principles (Figure 2).^9^

In a situation where the family physician may not be an expert on every clinical condition, symptom or learning need, it may be reassuring that one’s primary role is to facilitate learning rather than to have an answer for everything.

For many students, the family physician may also become something of a mentor, who coaches them on how to balance clinical and educational priorities or to cope with life events and stressors that threaten their learning.^13^ A good mentor may also enable personal development and life decisions.

### Information provider

The family physician is usually the most highly trained member of the clinical team and must be able to share their expertise and knowledge effectively.^13^

As a clinical teacher in the workplace, family physicians will need skills in conducting individual and small group teaching and assessment – for example, having a one-on-one educational meeting with a registrar, conducting a tutorial with medical students or giving a lecture as part of continuing professional development. In doing this they share their expertise and knowledge while maintaining a collaborative learning environment. Nowadays family physicians may also assist with online teaching and facilitate learning as well as provide information online. This may also require a set of skills suited to the virtual environment.

To be an effective information provider, the family physician must also understand how what they are providing fits into the intended learning outcomes of the broader curriculum and the rest of the educational programme. For staff they should align the information provided in continuing professional development with the key goals and priorities of the department of health or practice.

Family physicians may also require some educational planning skills if they are, for example, organising continuing professional development for the facility or registrar meetings throughout the year.

## Leadership as a clinical trainer and capacity builder

The ‘I–we–it’ model of leadership that is used in the training of family physicians also applies here.^17^

The effective educational leader must acquire appropriate educational theory and skills. This includes a shift towards more collaborative, adult and student–centred approaches to teaching. Teachers should embody the key principles of adult learning in their educational practice (Box 2). Most universities offer short courses for personal development, and the South African Academy of Family Physicians also runs a 5-day Training of Clinical Trainers course.^18^

BOX 2 Principles of adult learning.

**Adults need to know:** Adults need to know why they need to learn something. Help learners identify their learning needs prior to learning. Orientate your teaching to these learning needs.

**Adults have self-concept:** Adults need to be responsible for their own decisions, treated as capable of self-direction, respected as colleagues and encouraged to be independent and creative.

**Adults have experience:** Adults can learn from their lived experiences by reflecting, refining what they have learnt and applying it in a new situation. Reflection that challenges underlying assumptions may be transformative. As a teacher, you should facilitate such experiential learning cycles.

**Adults have readiness to learn:** Adults are ready to learn what will help them perform better in their roles or cope better in their life. Tailor teaching to their current roles and challenges.

**Orientation to learning:** Adults are motivated to learn things that will help them perform tasks better. Learning should be relevant and immediately applicable to real life.

**Motivation to learn:** Adults have more internal motivation to learn as they mature. Empower learners by building self-esteem and a sense of achievement.

*Source*: Knowles M. The adult learner: A neglected species. Vol. 4. Houston: Gulf; 1990^19^

Communities of practice^20^ are a group of people who share a concern or passion for what they do and learn how to do it better as they interact regularly. This is the environment we would like for our learners; such relationships are built on respect. Good relationships are critical to building a supportive organisational environment, particularly with the medical or clinical managers and senior health professionals. Good teachers are characterised as supportive, empathic, challenging, available, approachable and appreciative in their relationships with learners.^9^ Good relationships are also needed with educational institutions and key faculty members if they are placing students in your facility.

Finally, family physicians need to understand the key structures, procedures and processes that impact the learning environment at their facility. For example, are registrars rotated to different units in a way that addresses their learning needs as well as service delivery, is dedicated time for studying or learning incorporated into their schedule, are appropriate staff members selected for in-service training and are there mechanisms for supporting them to implement new skills on their return? Part of leadership is helping to shape and improve these processes within the facility.

## Conclusion

Family physicians have important roles as clinical trainers and capacity builders in the workplace. Learning is important for both staff and students. The organisational environment is an important determinant of the individual’s ability to learn or train others. The effective clinical trainer is a good role model as a clinician and educator, is a facilitator of learning as well as a mentor, and provides information through their clinical teaching. The family physician should provide leadership by ensuring that they have the necessary knowledge and skills, by building effective relationships and by influencing key organisational procedures and processes to support learning.
